# Apoptosis and apoptotic body: disease message and therapeutic target potentials

**DOI:** 10.1042/BSR20180992

**Published:** 2019-01-18

**Authors:** Xuebo Xu, Yueyang Lai, Zi-Chun Hua

**Affiliations:** 1 State Key Laboratory of Pharmaceutical Biotechnology, School of Life Sciences, Nanjing University, Nanjing 210023, China; 2 Changzhou High-Tech Research Institute of Nanjing University and Jiangsu TargetPharma Laboratories Inc., Changzhou 213164, China; 3 Shenzhen Research Institute of Nanjing University, Shenzhen 518057, China

**Keywords:** Apoptosis, Apoptotic bodies, caspase, Death receptors, drug design, intercellular communication

## Abstract

Apoptosis is widely known as programmed cell death eliciting no inflammatory responses. The intricacy of apoptosis has been a focus of an array of researches, accumulating a wealth of knowledge which led to not only a better understanding of the fundamental process, but also potent therapies of diseases. The classic intrinsic and extrinsic signaling pathways of apoptosis, along with regulatory factors have been well delineated. Drugs and therapeutic measures designed based on current understanding of apoptosis have long been employed. Small-molecule apoptosis inducers have been clinically used for eliminating morbid cells and therefore treating diseases, such as cancer. Biologics with improved apoptotic efficacy and selectivity, such as recombinant proteins and antibodies, are being extensively researched and some have been approved by the FDA. Apoptosis also produces membrane-bound vesicles derived from disassembly of apoptotic cells, now known as apoptotic bodies (ApoBDs). These little sealed sacs containing information as well as substances from dying cells were previously regarded as garbage bags until they were discovered to be capable of delivering useful materials to healthy recipient cells (e.g., autoantigens). In this review, current understandings and knowledge of apoptosis were summarized and discussed with a focus on apoptosis-related therapeutic applications and ApoBDs.

## Introduction

Apoptosis is a highly regulated process of cell death. Unlike necrosis which is a traumatic version of cell death, apoptosis is a rational and active decision made to sacrifice specific cells for the greater benefits of the organism. It is a normal physiologic process routinely carried out in multicellular organisms [1]. While many enigmas still remain in relevant research areas, it has been well-documented today that apoptosis confers advantages to multicellular organisms in a coordinated manner whereby organisms maintain homeostasis and fine-tune life cycle [2]. Its importance, both to multicellular organisms and to researchers, can be inferred from various biological responses and changes pertaining to apoptosis, e.g., embryonic development, cell renewal and turnover, and externally induced cell death (chemicals, radiations, etc.). One widely exploited function of apoptosis in the biomedical field is obliteration of cancer cells in response to extrinsically applied apoptosis-inducing stimuli such as small-molecule drugs. With apoptosis in the research spotlight, the profound therapeutic potential of apoptosis has enabled researchers to develop promising therapeutic solutions focusing on voluntary death of aberrant cells. A spectrum of drugs and therapies exploiting apoptosis has been proven effective against diseases. Funds and research efforts are being extensively invested in apoptosis-based research and clinical trials.

One notable feature of apoptosis is it exerts its effects mainly through action of a type of serine proteases known as caspases. A death signals are relayed through signaling pathways which ultimately lead to activation of caspases responsible for the execution of cell destruction [3]. Both external and internal stimuli, coupled to extrinsic and intrinsic apoptosis pathways, can initiate apoptosis. Despite many dissimilarities in different pathways, they converge to form apoptotic bodies (ApoBDs) which are eventually engulfed by phagocytes in a process known as efferocytosis [4]. Traditional views regard efferocytosis as the end point of apoptosis and therefore it biochemically concludes the life of morbid cells, whereas accumulating evidence converge to implicate on transfer, recycling and even reuse of materials packaged in ApoBDs [5]. A substantial part of current interest in apoptosis-related researches lies in apoptotic body formation. Apoptotic cells can also be effectively phagocytosed as a whole. In this review, current knowledge about apoptosis is discussed with a focus on therapeutic applications and ApoBD.

## Apoptosis and its involvements *in vivo*

Many forms of cell death exist, most of which can be triggered by various stimuli and physiological conditions. Among them, apoptosis and necrosis have been a well-known duo frequently being compared. In 1965, John Foxton Ross Kerr at University of Queensland made the first distinction between apoptosis and necrosis. Further refinements aiming to better interpret apoptosis followed. But it was not until 1972 when the term ‘apoptosis’ was first proposed by Kerr et al. [6]. Apoptosis has attracted much interest due to its intricate nature and diverse roles in maintaining a healthy and self-sustainable biological entity. Necrosis, on the other hand, is a form of cell injury in response to acute external damages and trauma, resulting in passive cell death which elicits inflammatory response [7]. Comparisons between apoptosis and necrosis reflect the unique features of each other and help researchers to elucidate their respective biological significance.

In cell cycle, apoptosis acts as a fail-safe measure that ensures fidelity and quality of proliferation. While certain degrees of genetic variation are allowed and favored for evolution, reproduced cells with extensive genetic errors and cellular damages are subjected to apoptosis. A key player in the cell cycle machinery, *p53*, initiates apoptosis in certain cell types. *p53* is an extensively studied tumor suppressor. Overwhelming evidence points to its exceeding importance in prevention of cancer development. The p53 tumor suppressor gene is most frequently mutated (mutated in over 50% of all human cancers) in cancer cells [8], rendering the restrictive mechanism ineffective. Tumorigenesis is likely to commence when the *p53*-based preventive system malfunctions or loses its function completely.

Stimuli such as DNA damage, hypoxia, and expression of certain oncoproteins (e.g., Myc, Ras) activates the *p53*-dependent apoptotic pathway [4]. Once committed, *p53* paves way for apoptosis by activating pro-apoptotic factors (e.g., Bax) while suppressing antiapoptotic factors (e.g., Bcl-2) [9]. As a well-known tumor suppressor, *p53* has been recognized for its critical function to initiate apoptosis in cell cycle, along with the ability to induce cell arrest and DNA repair in recoverable cells. There are many other cell cycle regulators besides *p53* that can influence apoptosis (e.g., pRb, p21). Nevertheless the mechanistic details of apoptosis in cell cycle are beyond the scope of this review.

Many researches highlighted the importance of apoptosis in the self-defense mechanism, or in other words, the immune system. The immune system is in charge of defensing the host against an array of external pathogens. Apoptosis is an integral part of the immune system where it facilitates to maintain a homeostasis of the immune system. For example, apoptosis is burdened with the responsibility to regulate immune responses, i.e., to induce death of T and B cells at certain time point to limit an immune response because a prolonged response would otherwise be deleterious to self. Second, the immune system depends upon apoptosis to eliminate unneeded T and B cells to be functionally mature [10]. For example, immune cells targeting self-antigens must be killed by apoptosis to prevent an attack on self. Or B cells that fail to generate antibodies of higher affinity for antigens are subjected to apoptosis as well. Lastly, cytotoxicity of certain types of cells (i.e., cytotoxic T lymphocyte and natural killer cells) is conferred by apoptosis. The well-coordinated killing protocol allows these cells to destroy target cells with themselves remaining intact. Cytotoxic T lymphocyte (CTL) can induce death in target cells through two pathways, one of which involves perforin and granzymes. Perforin and granzymes are contained within the granules excytosed from the CTLs in a directed manner. T cell receptors on CTL help to recognize a target cell (e.g., a cell infected by virus) and unload the granules on the surface of the target cell. Perforin, which is a protein capable of forming pores on the surface of cells, is released in a degranulation process and aid the entry of granzyme into the cell by punching holes on the cell surface [11]. Granzyme, which is also a serine protease, is key to DNA degradation associated with apoptosis in the target cells [12]. Distinct from the extrinsic and intrinsic pathways of apoptosis, perforin–granzyme-mediated apoptosis is exclusively employed in cytotoxic killing mediated by T cells.

Apoptosis plays an indispensable and irreplaceable role both under physiological and pathological conditions. Anomalies in apoptosis have become a major field of interest to researchers and are associated with a broad spectrum of pathological conditions, e.g., developmental defects, autoimmune diseases, cancer, etc. Some diseases pertain to deficiency of apoptosis while others pertain to its redundancy. For example, one of the hallmarks of cancer is evasion of apoptosis, meaning insufficient apoptosis overwhelmed by the limitless replicative potential of cells [13]. On the other hand, too much apoptosis is linked to certain pathological conditions such as acquired immune deficiency syndrome (AIDS). AIDS is a type of autoimmune disease caused by human immunodeficiency virus (HIV) infection [14]. HIV infects it host through binding to CD4 receptors on T cells, followed by subsequent internalization into T cells. Once inside the T cells, HIV increases the expression of Fas receptor which in turn incurs excessive apoptosis of T cells [15].

## Morphology and biology of apoptosis

Certain morphological changes exhibited by apoptotic cells have been well identified and documented. These changes include cell blebbing and shrinkage, nuclear fragmentation, condensation and fragmentation of genetic materials (chromatin and nucleosomal DNA), and formation of small vesicles known as ApoBDs. Cells undergo a characteristic shrinkage during the early stage of apoptosis. Cell size becomes smaller while the contents within the cells experience a denser packing. Another phenomenon known as pyknosis (irreversible condensation of chromatin) also occurs concomitantly at the early stage of apoptosis. These changes can be observed by light microscopy while electron microscopy can better visualize subcellular changes like pyknosis. Following the early stage of apoptosis, another phenomenon known as karyorrhexis, which refers to fragmentation of the nucleus, ensued. Karyorrhexis is accompanied by further blebbing of the plasma membrane that tears apoptotic cells into small ApoBDs. Current knowledge states that ApoBDs contain remnants of apoptotic cells (i.e., cytoplasm, organelles, and nuclear contents) where the contents of apoptotic cells are randomly distributed to each apoptotic body. Therefore, a specific organelle or a nuclear content may be or may not be present in a particular apoptotic body. These ApoBDs are then engulfed by phagocytes, e.g., neutrophils, macrophages, and dendritic cells (DCs), for final degradation. Phagocytosis marks the terminal step of an apoptotic cycle and it is believed that the essence of this step is to prevent spillage of hazardous materials packed within the apoptotic cells into the surroundings. The reason for forming ApoBDs has remained elusive. Details regarding ApoBDs will be discussed in the next section.

The biological mechanism of apoptosis is exceedingly sophisticated. A complete implementation of apoptosis involves interplay of a wide array of proteins and signal transducers as well as cascades of signaling pathways. Two major apoptosis pathways exist: extrinsic and intrinsic. The extrinsic pathway refers to receptor-mediated initiation of apoptosis. The death receptors in the extrinsic pathway are all anchored to the cell membrane by their transmembrane regions. Upon interaction with an extracellular ligand, membrane receptors relay death signals into intracellular space via their cytoplasmic death domains. Membrane receptors involved in apoptosis belong to the tumor necrosis factor (TNF) receptor superfamily, whose activation depends upon two major ligands: TNF and Fas. TNF and its receptors, namely TNFR-1 and TNFR-2, are responsible for initiating a major apoptosis pathway: the TNF pathway. The interaction between TNF and its receptors has been shown to relay death signal through recruitment of two adaptor proteins: TNF receptor-associated death domain (TRADD) and Fas-associated death domain protein (FADD), and effect programmed cell death through the action of caspases. TNF ligands form homotrimers which bind to TNF receptors on the membrane. Upon binding, adaptor proteins are recruited (TRADD, FADD, and RIP) to TNF receptors on the cytoplasmic side where death domains on the adaptor proteins interact with its counterparts on the receptors. FADD recruits its downstream interactor, procaspase-8 in this case, via homotypic interaction of the death effector domains (DED). Procaspase-8 then undergoes auto-cleavage to yield active caspase-8 which initiates the execution phase of cell death. Caspase-8 cleaves procaspase-3 via proteolytic cleavage to produce active caspase-3 responsible for the final execution of proteolytic degradation of a variety of intracellular proteins. Fas-mediated pathway undergoes a much similar signaling process. FasL (Fas ligand) triggers the pathway by binding to Fas receptors (also known as CD95). FADD associates with the Fas receptors and recruits caspase-8. Fas receptors, along with FADD and procaspase-8, form the death-inducing signaling complex (DISC).

A third extrinsic apoptotic pathway has been shown to be therapeutically exploitable, the TNF-related apoptosis inducing ligand (TRAIL) pathway. TRAIL, alternatively known as Apo2L, was first identified by its sequence homology to FasL [16]. It is a type II transmembrane protein containing an extracellular region which, upon proteolytic cleavage by protease, releases a soluble portion that acts as a ligand. Trimeric TRAIL binds to receptors on the membrane, namely DR4 and DR5, which then trigger intracellular signaling cascade similar to the Fas pathway [17,18]. The TRAIL-DR4/DR5 pathway is proposed to function in a wide range of physiological processes such as T cell activation and tumorigenesis. TRAIL is recognized as a tumor suppressor for its ability to exclusively induce apoptosis in malignant cells and xenografts, rendering TRAIL an ideal antitumor agent [16,19]. The TRAIL pathway also functions independently of *p53* which is frequently mutated in cancer cells, yet endowing TRAIL with another crucial therapeutic advantage [20]. However, a considerable number of cancers are resistant to TRAIL treatment [21]. Research on TRAIL has led to emergence of different variants of TRAIL possessing enhanced therapeutic efficacy than its precursor. Yao et al. first reported that RGD-TRAIL exhibited enhanced antitumor effect than wild type TRAIL in multiple tumor cell lines [22]. Receptor-specific TRAIL variants (i.e., specific to either DR4 or DR5) have also been shown to be more efficacious [23]. Another research group led by Dr. Hua recently engineered a novel variant of TRAIL constructed by linking Annexin V and TRAIL [24]. This variant, designated as TP8, showed surprisingly higher antitumor potency than RGD-TRAIL towards A549 cells.

The intrinsic pathway of apoptosis is characterized by non-receptor-mediated initiation and mitochondrial regulation. In the intrinsic pathway, stimuli directly generate intracellular signals which lead to biochemical changes within the cell. When a stimulus is present, it elicits disruption of the mitochondrial transmembrane that dissipates the membrane potential, rendering it more permeable. The stimulus also results in formation of mitochondrial permeability transition pore (MPT) on the outermembrane that channels pro-apoptotic factors into the cytosol [25]. Apoptosome cleaves procaspase-9 to yield active caspase-9 which in turn activates the effector caspase (i.e., caspase-3). Another group of proteins known as SMACs (small mitochondria-derived activator of caspases) bind to IAP (inhibitor of apoptosis proteins) following their release into the cytosol [26]. SMACs deactivate IAP, allowing apoptosis to proceed. Other inhibitors of IAP have been reported such as DIABLO and Omi [27,28]. These proteins engage in the caspase-dependent pathway of apoptosis. A second group of proteins known as apoptosis-inducing factors (AIFs) function in the caspase-independent pathway of apoptosis [29]. AIFs are anchored to the inner membrane of the mitochondria. Upon cleavage by calpain, which is a calcium-dependent protease, AIFs can translocate to the nucleus by nuclear localization signal (NLS) to induce DNA fragmentation and chromatin condensation [30].

Furthermore, research has shown that endoplasmic reticulum (ER) is also involved in apoptosis. Redundant accumulation of proteins and disturbance of calcium homeostasis within ER can trigger ER stress and therefore apoptosis. Mouse bearing caspase-12 knock-out are resistant to apoptosis, indicative of caspase-12’s involvement in ER-mediated apoptosis pathways [31]. Caspase-12, which is located on the membrane of ER, is essential to ER-mediated apoptosis. ER response elicits caspase-12 expression while translocating cytoplasmic caspase-7 to ER membrane where caspase-12 is activated and apoptosis ensues [32].

Caspases are pivotal components of apoptosis (Table 1). To date, two types of caspases have been defined, the initiator caspase and effector/executioner caspase. As previously mentioned, caspase-8 and -9 are initiator caspases while caspase 3 is effector caspase. Furthermore, other caspases such as caspase-2, -10, and -11 belong to the initiator category while caspase-6 and -7 belong to the effector category [36,37].

Members of the Bcl-2 protein family are responsible for regulation of apoptosis [38]. Two subgroups of this family perform opposite functions regarding apoptosis: they can be categorized as either pro-apoptotic or antiapoptotic (pro-survival). Table 2 illustrates the detailed classification and description of each member in the Bcl-2 family. In addition, *p53* has been shown to regulate members of the Bcl-2 protein family [39]. For example, *p53* can directly induce transcription of Bax which is a pro-apoptotic protein [40].

**Table 1:** Caspases in mammalian cells

Protease	Other names	Recognition sequence	Functions in apoptosis
Casp-1	ICE	YVAD	Cleavage of pro-interleukin, involved in death receptor-mediated apoptosis
Casp-2	ICE-1		Initiator or effector
Casp-3	CPP32, Yama, apopain	DEVD	Effector
Casp-4	ICErel-II, TX, TCH-2		Cleavage of pro-interleukin
Casp-5	ICErel-III, TY		Cleavage of pro-interleukin
Casp-6	Mch2	VEID	Effector
Casp-7	Mch3, ICE-LAP3, CMH-1		Effector
Casp-8	FLICE, MACH, Mch5		Initiator of death receptor-mediated apoptosis
Casp-9	ICE-LAP6, Mch6		Initiator
Casp-10	Mch4		Initiator of death receptor-mediated apoptosis
Casp-11	ICH3		Cleavage of pro-interleukin; initiator of death receptor-mediated apoptosis
Casp-12			Initiator in ER-mediated apoptosis
Casp-13			Also known as ERICE, functions unknown [33,34]
Casp-14			Filaggrin proteolysis and UV protection [35]
Casp-15			Unknown

**Table 2:** Bcl-2 family members

Protein encoded	Functions
Bcl-2	Apoptosis inhibitor, binds Bax and Bak
Bcl-x	L form inhibits, S form promotes apoptosis, binds Bax and Bak
Bcl-w	Apoptosis inhibitor
Bax	Pro-apoptotic, binds Bcl-2, Bcl-x_1_, and EIB1-9K
Bak	Pro-apoptotic or Apoptosis inhibitor, binds Bcl-2, Bcl-x_1_, and EIB19K
MCL-1	Apoptosis inhibitor
Bad	Pro-apoptotic, binds Bcl-2 and Bcl-x
Ced-9	Apoptosis inhibitor in nematode, homolog of Bcl-2
EIB19K	Apoptosis inhibitor in adenovirus, binds Bax and Bak

## Role of apoptosis in diseases

As previously described, apoptosis is a highly controlled physiological process in multicellular organisms. Research on molecular mechanism of apoptosis reveals that anomalies in apoptosis lead to many diseases. Therefore, factors involved in apoptosis regulation are of tremendous diagnostic and intervention value in curing diseases [41].

Defective apoptosis is associated with many types of illness including autoimmune diseases, neurodegenerative diseases bacterial and viral diseases, heart diseases, and cancer [42,43].

**Figure 1:**
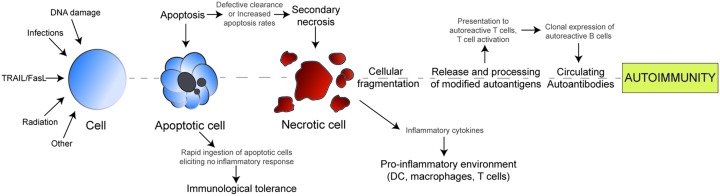
Defective clearance of apoptotic cells leads to inflammatory response and autoimmunity

Several reports have linked autoimmune diseases directly to dysregulated apoptosis and impaired clearance of apoptotic cells [44–49]. Rapid clearance of apoptotic cells is crucial to induce immunological tolerance and prevent inflammatory responses (Figure 1). Under physiological conditions, free apoptotic cells are rare in normal tissues [50–52]. However, inefficient ingestion of dying cells by phagocytes or increased rate of apoptosis can lead to secondary necrosis of free apoptotic cells, which induces secretion of pro-inflammatory cytokines. Autoantigens released from dying cells causes activation of T cell and expression of B cells, which generates autoantibodies and therefore autoimmunity (Figure 1). Many autoimmune diseases are consequences of impaired clearance of apoptotic cells such as systemic lupus erythematosus (SLE), connective tissue disease, and rheumatoid arthritis (RA) [53,54]. Defective apoptosis of immune cells can also cause autoimmune diseases such as multiple sclerosis and autoimmune lymphoproliferative syndromes [55,56]. Typically, the malfunctioning Fas/FasL apoptotic pathway interferes with normal apoptosis of lymphocytes leading to lymphocyte redundancy which in turn beget autoimmunity [57]. Furthermore, experimental results have shown that mouse lacking Fas on T lymphocytes showed lupus-like symptoms and lymphocyte hyperplasia [58].

While dwindled apoptosis of immune cells facilitates autoimmunity and fosters autoimmune diseases, too much apoptosis is as well a pathological condition [59]. Neurodegenerative diseases, such as Alzheimer’s disease, Huntington’s disease, and Parkinson’s disease are all characterized by excessive apoptosis of neurons. For example, Alzheimer’s disease is caused by accumulation of b-amyloids at lesion sites where b-amyloids induce abnormal apoptosis of neurons. Infections by HIV causes accelerated apoptosis CD4+ T cell, leading to onset of AIDS [60]. Myocardial infarction is caused by acute apoptosis of myocytes [61].

To summarize, impaired clearance of apoptotic cells, and too little or too much apoptosis are both disease-prone. Thus tuning apoptosis to a balanced state can serve to treat diseases [62]. If autoimmune lymphocytes cannot be cleared by apoptosis, these cells will attack autoantigens, triggering autoimmune diseases [63]. Research has shown that repeated administration of autoantigens to individuals with autoimmune diseases can cure them by inducing apoptosis and therefore clearance of lymphocytes that specifically attack these autoantigens. Members of the Bcl-2 protein family have become important drug targets because their abnormal expression causes many diseases [64]. Bcl-2, first member of its protein family and inhibitor of apoptosis, is pivotal to the occurrence and progression of cardiovascular diseases. A typical example is cardiac infarction which is caused by the lack of Bcl-2 expression and therefore excessive death of cardiomyocytes. Allowing overexpression of Bcl-2 in cardiomyocytes prevents apoptosis and has become an early stage therapeutic measure for cardiac infarction [65]. Evasion of apoptosis has become a prerequisite for occurrence of tumor. Some tumors, like follicular lymphoma, are related to elevated expression of Bcl-2 which can be counteracted by RNA interference and treatment by antisense oligonucleotides. Topoisomerase inhibitors, antimetabolic agents and alkylating agents belong to the class of cytotoxic drugs whose efficacy is realized through apoptosis induction of tumor cells [59]. For tumor cells expressing Fas, anti-Fas can specifically target these cells and induce rapid cell death. However, Fas is expressed on normal cells including peripheral while blood cells, T cells, and hepatocytes, therefore restricting the application of anti-Fas agonists. For apoptosis induced by cerebral ischemia, such as suffocation of newborns and cerebral ischemia in elderlies, along with Alzheimer’s disease, retinal degeneration, transplant rejection, and neurodegenerative diseases are all caused by excessive apoptosis. Therefore, inhibiting caspases is an effective therapeutic plan [66]. Cells undergo complex signaling pathways from apoptosis initiation to execution, involving a large network of proteins whereby blocking of any grid in the network can achieve therapeutic purpose [67]. For example, nerve growth factors inhibit apoptosis and apparently suit therapeutic needs to diseases of extensive apoptosis; or increasing Bcl-2 expression which lowers neuron’s sensitivity to apoptotic stimuli can inhibit pathological apoptosis of neurons in response to neurotoxic factors. Besides, small-molecule inhibitors of caspases, e.g., Z-VAD-FMK, has been proven useful in treating ALS in animals [68]. For AIDS treatment, antiretrovirus drugs are major players in the market despite their low efficacy and severe side effects. If the therapeutic strategy shifts its focus to shielding T cells from apoptosis and combines T cell protection with retrovirus destruction, the outcome will become much more satisfactory. Moreover, chronic myocardial ischemia and hypoxia cause apoptosis of cardiomyocytes and cardiac fibroblasts, which is preventable and treatable by blocking apoptosis [69]. In a word, designing treatment strategy based on the role of apoptosis in diseases associated with apoptosis anomalies holds great promises in restoring health and therefore is of critical research and application values (Table 3).

**Table 3:** Apoptosis-based drugs and therapies

Molecular target	Reagent	Principle	Experimental effects	Clinical trial/status	Company/reference
TRAIL receptors	HGS-ETR2	Agonistic TRAIL-R2 mAb	Apoptosis induction in tumor cell lines	Phase 1, up to 10 mg/kg every 2 weeks with minimal toxicity	[70]
	TRA-8	Agonistic TRAIL-R2 mAb	Apoptosis induction in tumor cell lines	Preclinical	Sankyo Co., Ltd.
	Dulanermin	Recombinant forms of TRAIL	Dulanermin plus bevacizumab was well tolerated with no occurrence of DLTs and demonstrated antitumor activity in NSCLC	Phase 2, 8 mg/kg/d for 5 days and 20 mg/kg/d for 2 days every 3 weeks in combination with PCB	[71]
	Mapatumamab	Agonistic TRAIL-R1 mAb	Safe and has promising clinical activity in patients with follicular lymphoma	Phase 1b/2	[72]
CD95/Fas	APG101	Target CD95 ligands	Restore immune function while effectively inhibiting tumor cell growth	Phase 2	ApoGenix
TNF	HUMIRA (Adalimumab)	Recombinant human IgG1k mAb against TNF-α	Inhibition of TNF-α	FDA approved for RA, psoriasis, ankylosing spondylitis (AS), Crohn’s disease	CAT/Abbott
	Recombinant TNF-α	Combination of TNF and chemotherapy	Apoptosis induction in tumor-associated blood vessels	Approved for isolated limb perfusion therapy in melanoma	[73,74]
	Infiximab (Remicade)	Mouse/human TNF-α antibody	Anti-inflammatory, induces also apoptosis in macrophages	FDA approved for RA and Crohn’s disease	Centocor/ScheringPlough
	Enbrel (Etanercept)	Recombinant TNF-R2/IgG fusion protein	Anti-inflammatory in RA, Crohn’s disease and other inflammations	Approved for US, some patients with severe side effects (infections, neurologic and hematologic disorders)	Amgen/Wyeth
				For RA and AS	
Pan-caspase	IDN-6556	Peptidomimetic irreversible caspase inhibitor	Antiapoptotic, anti-inflammatory, and antifibrotic in models of liver damage	Phase 2 started for chronic HCV infection, phase 2 opened for HBV infection and ischemia/reperfusion injury of liver transplants	Idun
				Phase 2 in compensatory and decompensated cirrhosis patients	
	MX1013	Dipeptide pan-caspase inhibitor	Prevents apoptosis in animal models of myocardial infarct, stroke, and acute liver failure	Preclinical, developed for myocardial infarct, stroke, acute liver failure	Maxim
	RGD peptides	Caspase activators	Apoptosis induction in tumor cell lines	Preclinical	Merck-Frosst, Maxim
Caspase-1	VX-740 (Pralnacasan)	ICE inhibitor	Anti-inflammatory in rheumatoid and osteoarthritis models	Phase 2, RA patients showed anti-inflammatory effects	Vertex/Aventis
Caspase-3	Immunocasp-3	Cell-permeable HER2 mAb fused to caspase-3	Growth inhibition in nude mice xenograft models containing PSMA-overexpressing LNCaP cells	Preclinical	[75]
Caspase-6	Immunocasp-6	Cell-permeable HER2 mAb fused to caspase-6	Growth inhibition of HER2-positive tumors in a mouse xenograft model	Preclinical	[76]
Caspase-9	FKBP12/caspase-9 fusion protein	Chemically inducible dimerization of caspase-9	Antiangiogenic in mouse models upon induction of caspase-9 dimerization	Preclinical	[77]
IAPs and SMAC	Embelin	Herbal cell-permeable XIAP inhibitor	Binds to XIAP BIR3, activates caspase-9, induces apoptosis in XIAP-overexpressing cells	Preclinical	[78]
	AEG35156/GEMR640	XIAP antisense oligonucleotide	Exhibits antitumor activity alone or in combination with chemotherapeutics in cancer xenograft models	Phase 1, phase 1/2 study of patients with relapsed/refractory AM	Aegera/Hybridon [79]
	SM-406	Antagonists of IAP proteins	Induces rapid degradation of cellular cIAP1 protein and inhibits cancer cell growth in various human cancer cell lines	Phase 1	[80]
	GDC-0917	Cell-permeable inhibitor of XIAP, cIAP-1 and cAIP-2	Potentiates apoptosis in combination with TRAIL and TNF, lead structure for development of IAP antagonists	Phase 1	Genentech
	LCL161	Antagonists of IAP proteins	Oral administration of LCL161 inhibits tumor growth in a mouse model of multiple myeloma142\143	Phase 2, neoadjuvant clinical trial of this weekly combination in triple negative breast cancer is in progress	Novartis
	APG-1387	Small-molecule IAP inhibitors	Promote the process of apoptosis by blocking the activity of IAPs	Advanced solid tumors, hematologic malignancies in clinical trials	Ascentage Pharma
Survivin	LY2181308	Survivin antisense construct	Preclinical studies show antitumor activity in a broad range of cancers	Phase 1	ISIS, Eli Lilly
Antiapoptotic Bcl-2 members	ApoG2	Targeting Mcl-1	Induced apoptosis and displayed promising results in different types of lymphomas	Preclinical	Ascentage Pharma
	ABT-263	Inhibit Bcl-2, Bcl-XL, and Bcl-w	Orally efficacious in an established xenograft model of human small cell lung cancer, inducing complete tumor regressions in all animals	Phase 1 in small cell lung cancer and hematological malignancies	[81]
	AT-101	A potent inhibitor of the antiapoptotic Bcl-2 family members, Bcl-2, Bcl-XL, and Mcl-1	Against B-cell lymphomas and displayed a synergistic effect when sequentially combined with 4-HC in diffuse large B-cell lymphoma	Phase 2	[82]
	Obatoclax	Inhibit Mcl-1 and antagonize Mcl-1-mediated resistance	Induce Bax-mediated apoptosis in cholangiocarcinoma	Phase 3 trial (NCT01563601) for Obatoclax in combination with Carboplatin and Etoposide compared with chemotherapy arm alone is underway in naïve patients with advanced-stage small cell lung cancer	Gemin X
	Genasense	Bcl-2 18-mer-antisense oligonucleotide	Kills drug-resistant chronic lymphocytic leukemia (CLL) cells, delays development of fatal lymphoma in mice, increases dacarbazine effectiveness in melanoma models	Phase 3: FDA fast-track status for melanoma, multiple myeloma, CLL. Phase 3 for non-small cell lung cancer, phase 2 for hormone-refractory prostate cancer	Aventis/Genta Inc.
	Venetoclax	BCL-2 inhibitor	Treatment of patients with CLL who have the 17p deletion mutation (del 17p) and who have previously received at least one therapy	April 11, 2016 approved by the US FDA	AbbVie
	APG-1252	Bcl-2 / Bcl-XL inhibitors	Selectively inhibit the Bcl-2 protein family members Bcl-2 and Bcl-XL to restore tumor cell apoptosis, thereby killing the tumor, is intended for the treatment of small cell lung cancer and other solid tumors	Phase 1	Ascentage Pharma
Pro-apoptotic Bcl-2 members	Pazopanib	Inducing PUMA expression	Effective antineoplastic agents that show promising clinical activity in a variety of carcinoma	Preclinical	[83]
p53	INGN201	p53-expressing adenovirus	Apoptosis induction in tumor cell lines and xenograft models	Phase 3 for head and neck cancer, clinical trials for other advanced solid tumors	Invitrogen Therapeutics
	SCH58500	p53-expressing adenovirus	Apoptosis induction in tumor cell lines and xenograft models	Phase 3 for advanced ovarian cancer	Schering-Plough
	ONYX-015	p53 delivery with mutant adenovirus	Virus demonstrates significantly greater antitumor activity against mutant p53 tumors *in vivo*	Phase 2/3 for combination therapy of advanced squamous cell cancer; phase 1/2 trials for several other cancers	Onyx
	C-terminal p53 peptides	Stabilization of wt and reactivation of mutant p53	Restore transactivation and growth-suppressing function of mutant p53	Preclinical	Aventis
	Nutlins	Imidazoline derivatives that antagonize p53/Mdm2 interaction	Drugs bind to the p53 pocket of Mdm2 and inhibit protein interaction *in vitro* and *in vivo*	Preclinical lead compounds	Roche
				Early clinical trials for blood cancers	
	Chalcones	Small-molecule antagonist of p53/Mdm2 interaction	Compounds with presumably insufficient specificity	Preclinical	[84]
	Small peptide compounds	Small-molecule antagonist of p53/Mdm2 interaction	High-affinity peptide that stimulates the p53 pathway	Preclinical	[85]
	APG-115	Small-molecule inhibitors that target the MDM2-p53 protein interaction	MDM2-p53 inhibitor, potently activates p53-regulated apoptosis. For sarcoma, primary liver cancer, primary gastric cancer and other tumors can form a highly effective inhibition, animal test administration allows tumor tissue completely disappear	Phase 1	Ascentage Pharma
	RG7388	p53-MDM2 inhibitors	Selectively binds to the p53 site on the MDM2 surface, isolating p53 from MDM2, resulting in activation of the apoptotic program following P53 stabilization, thereby killing cancer cells	Phase 3 of acute myeloid leukemia	Roche

Companies: Abbott Laboratories (www.abbott.com), Abbvie Inc. (www.abbvie.com), Aegera Therapeutics Inc. (www.aegera.com), Ascentage Pharma (www.ascentagepharma.com), Amgen (www.amgen.com), ApoGenix (www.apogenix.de), Aventis (www.aventis.com), CAT (Cambridge Antibody Technology, www.cambridgeantibody.com), Centocor (www.centocor.com), Eli Lilly and company (www.lilly.com), Gemin X Biotechnologies (www.geminx.com), Genentech (www.gene.com), Genta Incorporated (www.genta.com), Hybridon (www.hybridon.com), Idun Pharmaceuticals, Inc. (www.idun.com), Invitrogen therapeutics (www.invitrogen.com), ISIS Pharmaceuticals (www.isispharm.com), Maxim Pharmaceuticals (www.maxim.com), Merck-Frosst Canada & Co. (www.merckfrosst.ca), Novartis (www.novartis.com), Onyx Pharmaceuticals (www.onyx-pharm.com), Roche (www.roche.com), Sankyo Co., Ltd. (www.sankyo.co.jp), Schering-Plough (www.sch-plough.com), Vertex Pharmaceuticals, Inc. (www.vpharm.com), Wyeth (www.wyeth.com).

## ApoBD and the formation of ApoBD

Clearance of apoptotic cells has been a major field of study in apoptosis-related researches. Efficient and timely clearance of apoptotic cells is crucial to avoid immune response to autoantigens and prevent perturbation to ambient cells and tissues [86]. Majority of cells undergoing apoptosis are eliminated by phagocytes in the form of small vesicles known as ApoBDs. ApoBDs are relatively large vesicles compared with exosomes and microvesicles. ApoBDs have a diameter of 800–5000 nm while exosomes and microvesicles have a diameter of 30–100 and 50–1000 nm, respectively [87,88]. Furthermore, ApoBDs contain DNA, RNA, and proteins, similar to exosomes. However, the only marker of ApoBDs discovered so far is phosphatidylserine (PS) [89]. Although definitive answer to the purpose of ApoBDs is currently absent, it is proposed that breakage into ApoBDs contribute to more efficient clearance of apoptotic cells and are important in controlling immune responses [90].

**Figure 2:**
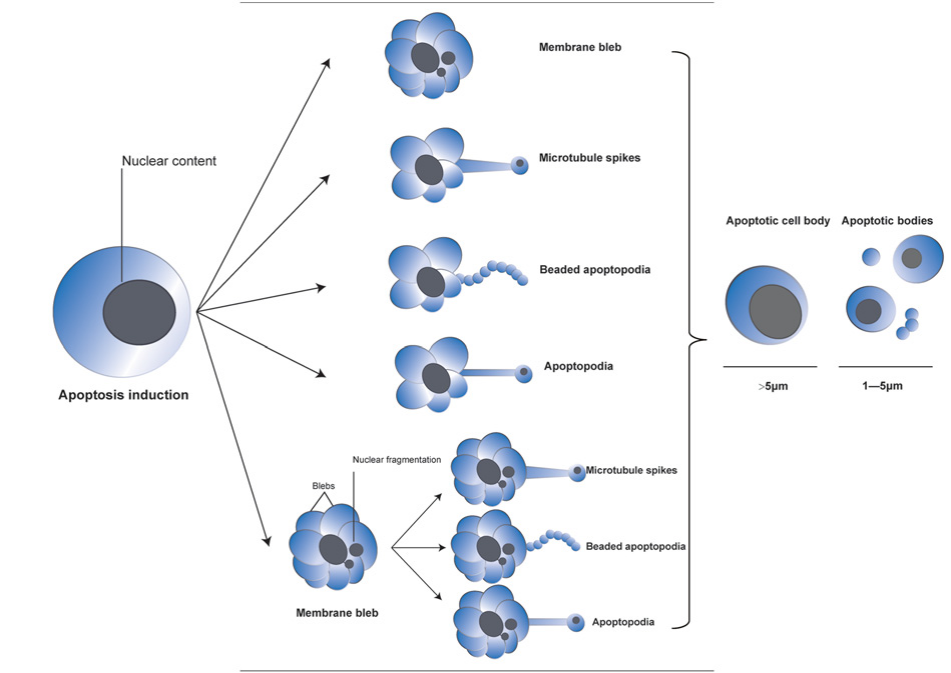
Different ways of cell disassembly into ApoBDs Patterns of apoptotic breakage into ApoBDs are illustrated in this figure. Majority of cells employ membrane blebbing, while some cells exhibit other types of membrane protrusions such as microtubule spikes, apoptopodia, and beaded apoptopodia.

Breakage of apoptotic cells into ApoBDs was previously believed to be a random process until a recent discovery revealed that a well-coordinated process is responsible for the formation of ApoBDs. As shown in Figure 2, one of the earliest and most identifiable morphological changes is deformation of cells that appears as membrane blebbing. Formation of blebs is a result of increased hydrostatic pressure within the cell following contraction mediated by actomyosin [91]. Notably, apoptotic blebs are distinct from necrotic blebs, which are generally larger, independent of actomyosin contraction, and are generated after membrane permeabilization [92]. Repeated process of blebbing and retraction of apoptotic cells gives rise to formation of ApoBDs packed with organelles and other cellular contents such as chromatin [93]. Another phenomenon known as apoptotic volume decrease (AVD) is also required for apoptotic body formation [94]. Cytoskeletal disruption which inhibits AVD halts the formation of ApoBDs [95]. AVD is an early event occurring concomitantly with membrane blebbing, leading to shrinkage of apoptotic cells. It is noteworthy that apoptotic blebs are different from necrotic blebs in many ways [96].

It is now assumed that membrane blebbing is a prerequisite for apoptotic body formation [97]. However, different cell types exhibit different forms of membrane deformation such as membrane protrusions including microtubule spikes, apoptopodia, and beaded apoptopodia [89]. For example, neurons and some epithelial cells use microtubule spikes, instead of membrane blebbing, to form ApoBDs. Human jurkat T cells, primary mouse thymocyte, and fibroblasts forms apoptopodia following membrane blebbing, while apoptotic THP-1 cells and primary human neutrophils displayed beaded apoptopodia structure. It is notable that beaded apoptopodia appears to be the most efficient way of producing ApoBDs, generating approximately 10–20 ApoBDs at the same time [89]. Beaded apoptopodia represents a unique mechanism of rapid formation of ApoBD. Interestingly, beaded apoptopodia and formation of ApoBDs were observed in neutrophils genetically engineered to be incapable of membrane blebbing, providing direct evidence that membrane blebbing is not required for formation of ApoBDs. Instead, other processes alone, or together with membrane blebbing, can promote disassembly of apoptotic cells and formation of ApoBDs. However, the detailed mechanism that ultimately rips cells into single ApoBD is still unclear. Researchers believe that a concerted effort of both intracellular and extracellular factors is required to shatter apoptotic cells into smaller vesicles and some unidentified force is there to separate membrane protrusions from the main cell body.

## The role of ApoBDs in cell clearance

Clearance of ApoBDs has been a focus in apoptosis-related researches. Phagocytes recognize ‘find-me’ signals released by apoptotic cells and ‘eat-me’ signals on the ApoBDs to engulf them [98]. Ingestion of cell corpses by phagocytes is termed efferocytosis as previously mentioned in the Introduction section. Efferocytosis is a four stage process that begins with locating the target cells [99]. ‘Find-me’ signals, which are soluble mediators released by the apoptotic cells, mediate attraction of phagocytes to the vicinity of apoptotic cells. Release of soluble signals starts at the very beginning of apoptosis where a gradient is set up by these mediators [86]. Signals are then recognized by receptors on the phagocytes, and triggers directional migration of phagocytes along the gradient towards ApoBDs. The second stage features ‘Eat-me’ signal recognition on ApoBDs. The ‘eat-me’ signals are represented by PS which becomes externalized and serves as signals for recognition and anchorage of phagocytes, allowing them to initiate the engulfing process [100]. The third stage is engulfment. Cytoskeletal rearrangement and modification of the phagocytes occur to enable ingestion of ApoBDs [101]. The last stage is digestion of cellular remnants through lysosomal degradation. The ‘eat-me’ signal PS can interact with a calcium phospholipid binding protein, Annexin V. Annexin V is a widely expressed protein belonging to the annexin superfamily. It is largely involved in blood coagulation due to its ability to bind PS [102]. Therefore, Annexin V derivative has been developed to identify and image apoptosis [103–105].

Although ApoBDs can readily be engulfed by phagocytes, intact apoptotic cells can be engulfed just as effectively [96]. In fact certain cell types do not fragment and form ApoBDs. However, *in vitro* studies showed that suppression of apoptotic blebbing, therefore formation of ApoBDs, impaired clearance of apoptotic cells by monocytes and macrophages [106,107].

## ApoBDs are biological entities carrying functional biomolecules

**Figure 3:**
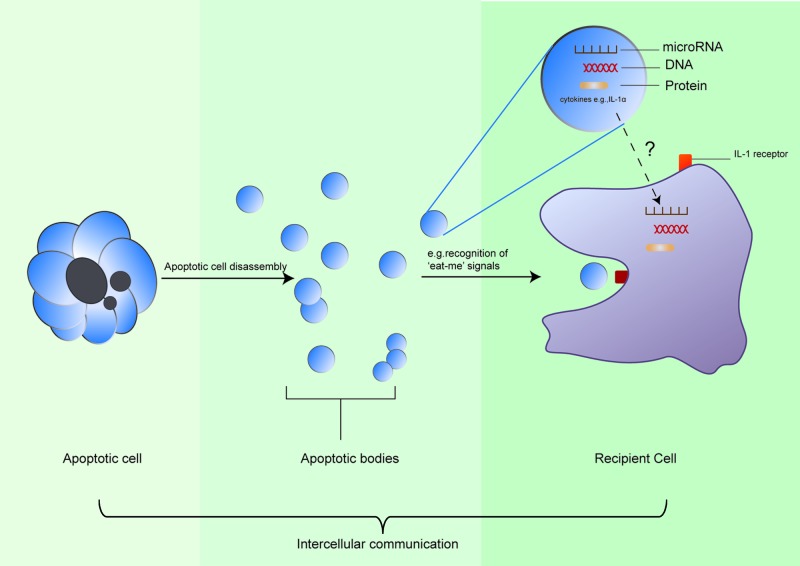
ApoBDs are implicated in intercellular communications DNA, RNA, proteins, and cytokines are contained within ApoBDs. Transfer of macromolecules are mediated by ApoBDs upon engulfment of ApoBDs by recipient cells, in which contents of ApoBDs are released. Appropriate molecules released by ApoBDs commence downstream biological response or signaling pathways in recipient cells, therefore achieving the purpose of intercellular communication.

Extracellular vesicles (e.g., exosomes and microvesicles) play a critical role in intercellular communications by transporting intracellular signaling molecules [108] Likewise, ApoBDs are vesicles that encapsulate residual ingredients of dying cells. Traditional views believed that apoptotic cells are phagocytosed to prevent deleterious impacts on the surroundings. A specific apoptotic body may contain a wide variety of cellular components, i.e., cytosol, degraded proteins, DNA fragments or even an intact organelle (Figure 3). An interesting question to ask is whether these cellular contents, are randomly distributed to each apoptotic body.

Recent studies have unraveled involvement of ApoBD in progression, metastasis, and formation of microenvironment of tumor. The discovery of ApoBD has offered new insights and explanations for physiological and pathological conditions. During the period of apoptosis, membrane blebbing promotes distribution of nuclear material into ApoBDs [109]. Horizontal transfer of DNA can occur between adjacent whereas different types of cells. For example, DNA packaged into lymphoma-derived ApoBDs was engulfed by surrounding fibroblasts, resulting in the integration of lymphoma-derived DNA into the fibroblast genome [110]. Transfer of oncogenes (h-ras and c-myc) by ApoBDs to recipient cells lacking p53 facilitates tumor formation [111]. Apart from DNA, ApoBDs of endothelial cells mediate transfer of miRNA (i.e., miRNA-126) to healthy endothelial cells, and induce expression of a chemokine CXCL12 in recipient cells. In mouse models bearing atherosclerosis, repeated administration of ApoBDs containing miRNA-126 ameliorate the condition, at which this therapeutic effect may be mediated through induction of CXCL12 expression in luminal cells of aortic root plaques and recruitment of endothelial progenitor cells to repair blood vessels [112]. Interestingly, DNA and RNA are packaged into ApoBDs of HL-60 cells, a type of granulocyte [113]. Although the molecular mechanism is to be elucidated, an accumulation of reports has shown that functional molecules (i.e., DNA, RNA, and proteins) can indeed be packaged into ApoBDs, and different type of molecules follow different signaling pathways and therefore produce varied biological outcomes.

Apart from nucleotides, proteins can be transferred by ApoBDs to phagocytes, such as macrophages and DC cells, for immunoregulation. ApoBDs of thymocytes from BALB/c mouse are enriched with autoantigens and pro-inflammatory factors, along with 142 different protein including histones, hsp90, and many immunoproteins [114]. Similarly, ApoBDs from lymphoblasts contain histones 1, 2A, 2B, 3, and 4, as well as autoantigen La/SSB 974. Shotgun proteomics identified 11 different proteins unique to ApoBDs of biliary epithelial cells as compared with their healthy counterparts [115]. These proteins include Annexin A6, hsp6, LDL receptor-related proteins. These proteins are implicated in many immune pathways, e.g., activation of NF-κB, ERK, and Notch pathways, and signaling pathways mediated by IL8 and CXCR2. Recent studies also reported a drastic difference in 1028 proteins [89]. Many research teams reported distribution of autoantigens into membrane vesicles and ApoBDs from a variety of cells (e.g., epithelial cells, T cells, cardiomyocytes) [116]. It is noteworthy that human primary monocyte-derived macrophage can engulf ApoBDs containing autoantigens, suggesting a possible route through which autoantigens can be transferred to professional phagocytes [117]. Furthermore, endothelial cells-derived ApoBDs contain precursor and processed pro-inflammatory factor IL-1α. ApoBDs containing IL-1α promote expression of chemokine IL-8 from healthy endothelial cells, thereby facilitating invasion of neutrophils into mouse peritoneum [118].

Contents of within the ApoBDs may be used for a number of purposes such as activation of immune system, recruitment of dying cells and regeneration of damaged tissues. Rubartelli et al. first reported that DC can selectively engulf ApoBDs [119]. DC is a type of phagocytes capable of engulfing ApoBDs rich in auto-antigens. It is first reported in 1998 by Albert et al. that DCs acquire antigens from ApoBDs and induce class I-restricted CTLs [120]. The same group later reported a continuation of this study that ApoBDs contain processed antigens that can be directly used by DCs for cross-presentation [121]. In bone marrows, dying osteocytes release ApoBDs containing receptor activator of nuclear factor kappa-B ligand (RANKL) to recruit replacement osteoclasts [122].

Schiller and co-workers observed that immunogenic molecules enter ApoBDs in the early stage of apoptosis before DNA fragmentation [123]. JAK1/STAT3 in hepatic stellate cells (HSC) pathway was activated through engulfment of ApoBDs derived from HepG2 cells, which also activated PI3K/Akt/NF-kB survival pathway to a lesser degree. Antiapoptotic proteins Mcl-1 and A1 were upregulated, leading to survival of HSC and spread of liver fibrosis [124]. Marin-Gallen et al. recently revealed therapeutic capacity of ApoBDs [125]. They reconstituted peripheral tolerance of type I diabetes by forced intake of in ApoBDs derived from *in vitro* cells by apoptosis-resistant DC cells. Therefore, these DC cells expressed lowered level of CD40 and CD86, and pro-inflammatory cytokines. As a result, autoimmunity against cells is reduced. These results suggest that ApoBDs can act as regulators at the cellular level and therefore possess conspicuous biological significance.

Furthermore, ApoBDs are carriers of contagious materials of pathogens. For example, ApoBDs of HIV-infected T cells can transfer proteins and genomic contents of HIV to adjacent epithelial cells where transcription and expression of viral proteins are furthered and assembly of HIV takes place [126]. Besides, ApoBDs of prion-infected neurons must be effectively eliminated to prevent spreading of prions and onset of prion diseases [127].In mouse models bearing tumor xenografts, ApoBDs were detected in the blood [128]. Moreover, murine fibroblasts lost contact inhibition and became tumorigenic after engulfing ApoBDs derived from cells transfected with oncogenes [129]. These results suggest that genetic information can also be transferred by uptake of ApoBDs. Tumor cells release ApoBDs to environment upon apoptosis induced by treatments, which promotes tumor invasion and metastasis [127,130]. Hence, ApoBDs can function as a ‘Trojan horse’ for infectious agents [131].

To conclude, ApoBDs are messengers containing legacy of its progenitors, whose destiny lies far beyond being ‘garbage bags’ as we previously assumed. They can be recycled to initiate an array of biological responses. Further understanding of ApoBDs is essential to complete delineation of apoptosis.

## Concluding remarks

The more efforts invested, the more we realize how complex the process of apoptosis is. It is a highly coordinated process of both morphological and biochemical change. Depending on the cell type and disassembly pattern, ApoBDs of different size and amount can be generated. Therefore, an obvious question can be asked: why the universal process of apoptosis can present itself in various ways? Why different cell types employ different ways of breakage? What controls the release, size and contents of ABs? By solving these mysteries, we become one step closer to complete understanding of programmed cell death under both physiological and pathological conditions. Lastly, it is encouraging that drugs that block or promote ApoBD formation have been advanced to the stage of clinical trials [132]. Therefore, therapies and drugs designed based on regulation apoptosis and ApoBD formation hold great promises in curing intractable diseases [133].

## Abbreviations

AIDS: acquired immune deficiency syndrome
AIF: apoptosis-inducing factors
ApoBDs: apoptotic bodies
AVD: apoptotic volume decrease
CLL: chronic lymphocytic leukemia
CTL: cytotoxic T lymphocyte
DC: dendritic cell
DED: death effector domains
ER: endoplasmic reticulum
FADD: fas-associated death domain protein
HIV: human immunodeficiency virus
HSC: hepatic stellate cells
PS: phosphatidylserine
RA: rheumatoid arthritis
TNF: tumor necrosis factor
TRADD: TNF receptor-associated death domain
